# Multiple Epiphyseal Dysplasia With Knee Joint Locking Symptoms Caused by Intra-articular Loose Bodies

**DOI:** 10.7759/cureus.58906

**Published:** 2024-04-24

**Authors:** Takaki Kitamura, Shuji Yamazaki, Takehiro Kijima, Yasuyuki Takamori, Eiichiro Watanabe

**Affiliations:** 1 Department of Orthopaedic Surgery, Chiba University, Graduate School of Medicine, Chiba, JPN; 2 Department of Orthopaedic Surgery, Fuji Orthopaedic Hospital, Fuji, JPN; 3 Department of Orthopaedics and Traumatology, Fuji Orthopaedic Hospital, Fuji, JPN

**Keywords:** locking symptom, matrilin-3, adult-onset, arthroscopic knee surgery, intra-articular loose body, multiple epiphyseal dysplasia

## Abstract

Multiple epiphyseal dysplasia (MED) is a congenital disease causing epiphyseal dysplasia in long bones. Herein, we report a case of a middle-aged man with bilateral knee joint locking symptoms who was diagnosed with multiple epiphyseal dysplasia caused by Matrilin-3 (MATN3) pathogenic variants and was successfully treated with arthroscopic loose body removal. A 48-year-old man has had bilateral knee pain since his twenties and underwent loose body removal of both knees in his thirties. He visited our hospital for worsening locking symptoms in both knees. Twenty years ago, his son had been diagnosed with suspected multiple epiphyseal dysplasia. Genetic and imaging testing confirmed his diagnosis of multiple epiphyseal dysplasia due to Matrilin-3 pathogenic variants. Arthroscopic loose body removal was performed, and the locking symptoms disappeared after surgery. Arthroscopic loose body removal was effective for the locking symptoms in a mild adult case of multiple epiphyseal dysplasias caused by Matrilin-3 pathogenic variants.

## Introduction

Multiple epiphyseal dysplasia (MED) is a relatively common congenital disorder that causes abnormal ossification of the epiphyses of long bones and joint deformities at a young age, primarily in load-bearing joints such as the hip and knee [1,2]. The documented incidence of MED spans from 1 in 10,000 to 1 in 20,000 individuals, and the hip joint is the most commonly affected, with the most frequent chief complaint in adult cases being pain due to osteoarthritis. MED is an autosomal dominant disorder with a variable phenotype depending on the causative gene and is primarily diagnosed in childhood [3]. The diagnosis of MED remains challenging due to inconsistencies in the symptoms and severity caused by the variety of causative genes. Matrilin-3 (MATN3) genetic mutations are often associated with mild symptoms, which may not be diagnosed until the patients begin to experience knee osteoarthritis in adulthood [4]. In surgical treatment, total knee arthroplasty (TKA) would be reasonable for elderly patients. For younger patients, arthroscopic surgery should be performed, including loose body removal or cartilage transplantation, depending on the symptoms, although reports on these procedures are limited. We present a case of a middle-aged man with bilateral knee joint locking symptoms who was diagnosed as MED with mutations in MATN3 by genetic testing.

## Case presentation

**Figure 1:**
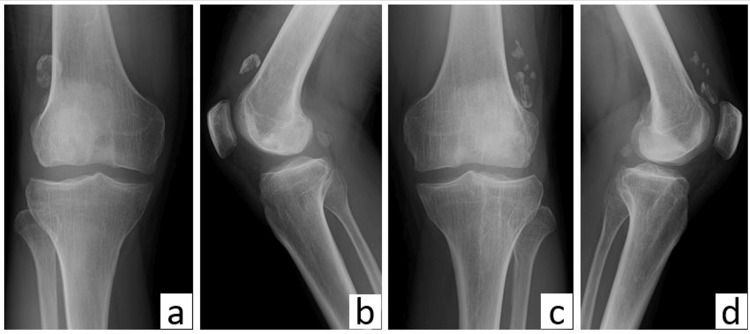
Plain radiographs of both knees prior to surgery (a) Anteroposterior and (b) lateral plain radiographs of the right knee; (c) anteroposterior and (d) lateral plain radiographs of the left knee.

The patient was a 48-year-old man with a height of 164 cm. At the age of 20, he presented with bilateral knee pain, and by the age of 30, he underwent loose body removal of both knees at another institution. However, the identification of the disease that necessitated surgery due to loose bodies in both knees at a young age was not achieved, and the locking symptoms of the knee recurred subsequently. He presented to our hospital two years ago with symptoms of bilateral knee pain and locking symptoms. He had no history of vigorous sports or trauma episodes on his knees. A radiographic examination revealed a flat intercondylar eminence in both his tibiae with multiple large, loose bodies (Figure 1).

**Figure 2:**
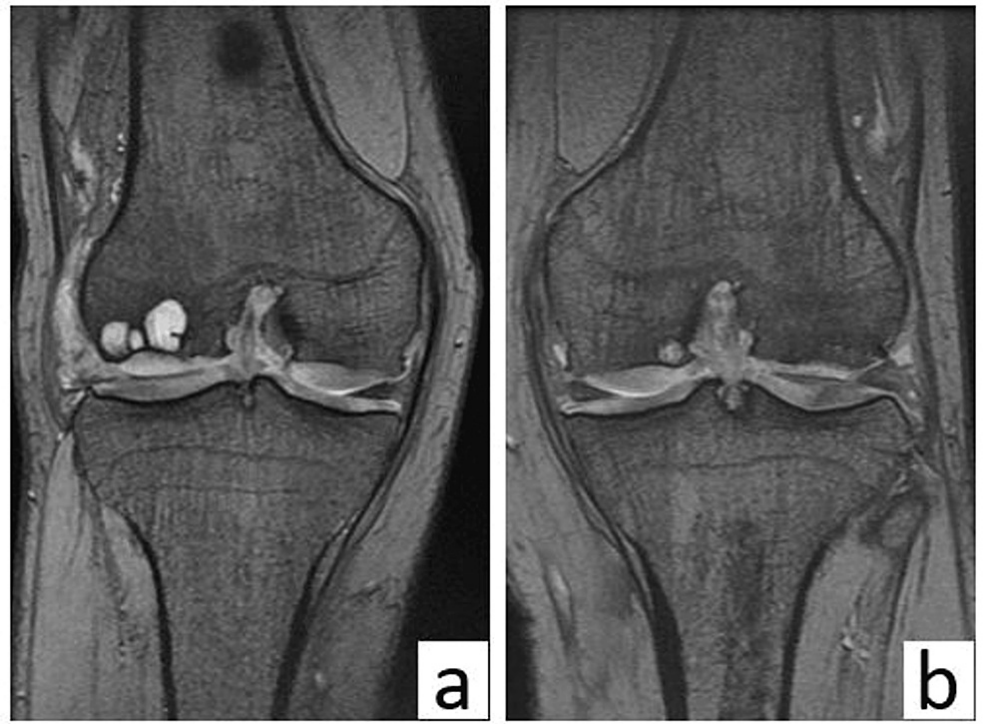
Magnetic resonance imaging of both knees prior to surgery (a) Coronal T2-weighted magnetic resonance image of the right and (b) left knees.

We suspected a skeletal disorder, but the patient was lost to follow-up. Two years later, he returned to our hospital for a comprehensive examination because his symptoms had exacerbated and had started to impair his ability to work as a carpenter. Magnetic resonance imaging revealed cartilage hypertrophy and multiple bone cysts in both knees (Figure 2).

His family history was noteworthy; he had a son who had been admitted to a children's hospital with suspected MED and a brother with a history of bilateral knee pain from a young age. Based on the radiological findings of the knee and the characteristic family history, we strongly suspected MED, even though there were no X-ray findings indicating MED in any joints other than the knee. As the patient was eager to receive a definitive genetic diagnosis of his condition due to the history of his son's surgery and his brother's similar symptoms, we conducted genetic testing following genetic counseling. Genetic testing confirmed the diagnosis of MED caused by an underlying mutation in the MATN3 gene. Given that MED is a genetic disorder, his definitive diagnosis not only facilitated his own management but also led to the diagnosis of his brother and son, enabling them to receive orthopedic consultation and lifestyle guidance for their symptoms.

**Figure 3:**
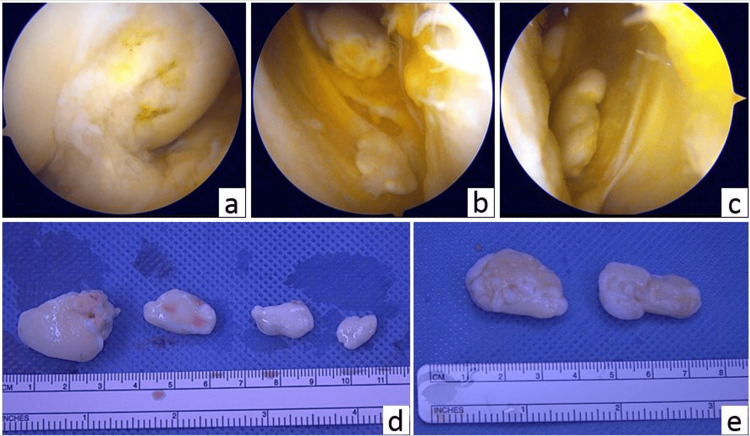
Arthroscopic images and extracted loose bodies (a) Flattened trochlear groove, and loose bodies in the lateral pouch of the (b) right and (c) left knees (arthroscopic images). Extracted loose bodies of the (d) right and (e) left knees.

As the patient desired to return to work as soon as possible, he underwent arthroscopic loose body removal and a partial lateral meniscectomy of both knee joints to alleviate his knee-locking symptoms. Multiple large, loose bodies were removed, and the locking symptoms were resolved (Figure 3).

He returned to work two weeks after the surgery. Three months following the operative procedure, the Lysholm knee assessment revealed a score of 77 for both knees, with responses indicating 'a catching sensation but no locking.' This outcome is presumed to arise from the consequences of residual meniscus injury and cartilage damage, with the prospect of further surgical intervention being evaluated based on the patient's pain levels.

## Discussion

Overall, we report the case of a middle-aged man presenting with bilateral knee-locking symptoms caused by multiple loose bodies caused by MED that were finally diagnosed genetically as MED caused by mutations in the MATN3 gene.

MED is frequently inherited in an autosomal dominant manner, and six causative genetic mutations have been identified to date [3]. MED induces symptoms of osteoarthritis in various joints, and several studies have indicated that the most frequent genetic aberration is an underlying mutation in the cartilage oligomeric matrix protein (COMP) gene, which frequently manifests with symptoms of osteoarthritis in the hip joint at an early stage [2]. However, in Japan, mutations in MATN3 are as prevalent as mutations in COMP [5]. In comparison to COMP mutations, MATN3 mutations are often correlated with milder symptoms, which may postpone diagnosis until the development of osteoarthritis [4]. Despite the lack of clear diagnostic criteria, radiographic evaluations of the knee joint are often beneficial for the diagnosis of MED and typically reveal findings such as flattened tibial intercondylar eminences, shallow femoral trochlear grooves, depression of lateral tibial plateaus, enlarged joint cavities, loose bodies, and genu valgum deformities [6,7]. In the present case, locking symptoms prompted the patient to seek medical attention, and knee joint radiographs demonstrated typical imaging characteristics of MED; however, other joint radiographs did not show any epiphyseal dysplasia. MED resulting from MATN3 mutations may present with milder symptoms restricted to the knee and may cause locking symptoms due to multiple loose bodies. Mutations in MATN3 may be more frequent than previously assumed, possibly due to the mild nature of knee symptoms that may be overlooked. If cases of mild MED, as demonstrated in the present case, are swiftly detected without fail, it becomes feasible to implement initial interventions like advising on lifestyle modifications, which could potentially slow the worsening of symptoms.

The initial management of adult cases of MED is conservative [3], but in most cases, osteoarthritis tends to progress, requiring surgical intervention. In cases of MED caused by mutations in MATN3, the knee joint is the second most commonly affected joint after the hip joint, with very few reports discussing knee joint surgery [6]. Even though surgical treatments are required, alternative procedures other than joint replacement are desirable, especially in young patients. Taketomi et al. reported that a case treated with an arthroscopic osteochondral autograft for a femoral chondral lesion showed good long-term results in terms of knee pain relief [8]. Regarding knee-locking symptoms, previous reports have indicated underlying loose bodies as well as a femoral posterior chondral flap as a possible cause [9]. In the present case, the knee-locking symptoms improved with the removal of loose bodies alone, and the patient was able to return to work early after surgery.

## Conclusions

The present cases suggest that the possibility of MED caused by mutations in MATN3 should be considered as a potential diagnosis in patients with knee joint locking symptoms caused by multiple loose bodies in adults without any traumatic or pathologic history. Moreover, the precocious identification of MED facilitates the initiation of prompt therapeutic measures, and genetic consultation becomes imperative when MED is conjectured in light of familial antecedents. The literature contains scant discourse on the application of arthroscopic surgery for the management of MED, underscoring the need for an augmented corpus of case studies to elucidate the efficacy of this therapeutic approach.
